# Effects of Environmental Stresses on Synthesis of 2-Phenylethanol and IAA by *Enterobacter* sp. CGMCC 5087

**DOI:** 10.3390/microorganisms12040663

**Published:** 2024-03-26

**Authors:** Ke Li, Senbiao Fang, Xiao Zhang, Xiaodi Wei, Pingle Wu, Rong Zheng, Lijuan Liu, Haibo Zhang

**Affiliations:** 1 College of Life Science and Technology, Inner Mongolia Normal University, Hohhot 010022, China; like@qibebt.ac.cn (K.L.); wxd15636786477@163.com (X.W.); wupingle2023@163.com (P.W.); 2 Qingdao Institute of Bioenergy and Bioprocess Technology, Chinese Academy of Sciences, Qingdao 266101, China; fangsenbiao@qibebt.ac.cn (S.F.); zhangx@qibebt.ac.cn (X.Z.); zhanghb@qibebt.ac.cn (H.Z.); 3 Shandong Energy Institute, Qingdao 266101, China; 4 Qingdao New Energy Shandong Laboratory, Qingdao 266101, China

**Keywords:** 2-PE, IAA, stress conditions, *KDC4427* expression, enzyme activity

## Abstract

2-Phenylethanol (2-PE) and indole-3-acetic acid (IAA) are important secondary metabolites produced by microorganisms, and their production are closely linked to the growth state of microorganisms and environmental factors. *Enterobacter* CGMCC 5087 can produce both 2-PE and IAA depending on α-ketoacid decarboxylase KDC4427. This study aimed to investigate the effects of different environment factors including osmotic pressure, temperature, and pH on the synthesis of 2-PE and IAA in *Enterobacter* sp. CGMCC 5087. The bacteria exhibited an enhanced capacity for 2-PE synthesis while not affecting IAA synthesis under 5% NaCl and pH 4.5 stress conditions. In an environment with pH 9.5, the synthesis capacity of 2-PE remained unchanged while the synthesis capacity of IAA decreased. The synthesis ability of 2-PE was enhanced with an increase in temperature within the range of 25 °C to 37 °C, while the synthesis capacity of IAA was not affected significantly. Additionally, the expression of *KDC4427* varied under stress conditions. Under 5% NaCl stress and decreased temperature, expression of the *KDC4427* gene was increased. However, altering pH did not result in significant differences in gene expression levels, while elevated temperature caused a decrease in gene expression. Furthermore, molecular docking and molecular dynamics simulations suggested that these conditions may induce fluctuation in the geometry shape of binding cavity, binding energy, and especially the *d_αC-C-_* value, which played key roles in affecting the enzyme activity. These results provide insights and strategies for the synthesis of metabolic products 2-PE and IAA in bacterial fermentation, even under unfavorable conditions.

## 1. Introduction

2-Phenylethanol (2-PE), an aromatic with delicate rose fragrance, is widely used in foods, cosmetics, and pharmaceuticals, and it is also a candidate for advanced biofuels [1,2,3]. It is widely present in the flowers and fruits of plants, fungi, and some bacteria, and it is well known due to its antimicrobial properties. Furthermore, it can serve functions in bacterial competitiveness [2,4,5,6,7,8]. Indole-3-acetic acid (IAA), also known as auxin, is one of the most important phytohormones and widely used in modern agriculture [9,10,11]. In addition to its role in regulating plant development and physiological processes, IAA also plays an important role in microbial physiology [12]. Currently, the synthesis and regulation of both 2-PE and IAA have been investigated independently in microorganisms [7,13]. Additionally, each of these compounds has been produced in various engineered microbial strains [14,15,16]. *Enterobacter* sp. CGMCC 5087 was isolated from sewage sludge of dye wastewater and classified as *Enterobacter* species. This strain is capable of de novo synthesizing 2-PE [17]. Recently, it has been shown that *Enterobacter* sp. CGMCC 5087 produces both 2-PE and IAA in an α-ketoacid decarboxylase KDC4427 dependent manner [8]. KDC4427 exhibits both phenylpyruvate decarboxylase (PPDC) and indolepyruvate decarboxylase (IPDC) activities, and can efficiently catalyze the decarboxylation of phenylpyruvic acid (PPA) and indolylpyruvate acid (IPyA) to form phenylacetaldehyde and indoleacetaldehyde, respectively. Subsequently, they are converted to 2-PE and IAA by acetaldehyde reductase and acetaldehyde oxidase, respectively [7,18,19,20]. 

A variety of studies have demonstrated that stressful environments can significantly affect the synthesis of metabolites [8]. When microorganisms encounter changes in environmental conditions or stressors, their metabolic state undergoes alterations [21]. This can occur through adaptations or changes in gene expression and enzyme activity in response to stressful conditions [22,23]. Studies have shown that appropriately increasing the temperature can enhance the influx of precursor substances into the synthesis pathway of 2-PE in plants such as *Camellia sinensis*, *tomato,* and *petunia*, thereby increasing the overall synthesis of 2-PE [24]. Additionally, the effects of low-temperature (10 °C, 15 °C, and 20 °C) fermentation on metabolite compositions of *Saccharomyces cerevisiae* were investigated. The results indicated that the levels of 2-PE, ethyl acetate, and other substances decreased as the temperature decreased [25]. Changes in pH can impact the growth and metabolic activity of yeast as well as enhance the volatility of aromas, such as 2-PE, through interactions with other stress conditions [26]. It was reported that the production of 2-PE is influenced by both temperature and pH, with the optimal cultivation conditions determined as 40 °C and pH 7.0 in a recombinant *Escherichia coli* strain. Alterations in fermentation conditions resulted in a decrease in 2-PE yield, underscoring the significance of different fermentation conditions for efficient synthesis of 2-PE [6]. Changes in pH can have an effect on the synthesis of IAA in bacteria [27]. When cultured in an acidic environment with a pH of 4.0, *Candida* sp. JYC072 exhibited a significantly higher production of IAA in comparison to that with a pH of 6.5. Conversely, under alkaline conditions with a pH of 9.0, although certain yeast species were able to thrive, IAA production was not observed [28]. Based on the above, it is evident that the growth environment significantly influences the synthesis of 2-PE and IAA by bacterial strains. However, further research is needed to fully describe the impact of different stress conditions on the synthesis of 2-PE and IAA.

In this study, we investigated the influence of various stress conditions on the production of 2-PE and IAA, as well as the synthesis capability of *Enterobacter* sp. CGMCC 5087. Furthermore, the expression of essential gene *KDC4427* involved in the synthesis of 2-PE and IAA were analyzed in related environment conditions. Finally, the catalytic mechanism difference under these conditions (NaCl, pH, and temperature), which affected the binding cavity volume, binding free energy, and distance *d_αC-C-_* between αC atom (PPA and IPyA) and C^-^ atom (thiamine diphosphate, ThDP), were elaborated in detail through molecular docking and molecular dynamics (MD) simulations. Further insights are expected from this study about how environment conditions regulate the production of 2-PE and IAA, which will be beneficial to guiding the effective production of 2-PE or IAA in engineered strains. Therefore, this work enriched our understandings of 2-PE and IAA synthesis in different environmental conditions in bacteria and provided insights into the production of these metabolites by the bacterial fermentation process.

## 2. Materials and Methods

### 2.1. Bacterial Strain and Culture Conditions

The *Enterobacter* sp. CGMCC 5087 was cultured at 37 °C in Luria broth (LB) medium unless otherwise indicated. For analysis of the effects of different environmental stresses on the growth and products synthesis in *Enterobacter* sp. CGMCC 5087, the bacteria were cultured overnight at 37 °C in a rotary shaker at 180 rpm/min in LB medium. Subsequently, the bacterial cells were transferred to 5 mL of LB medium under stress conditions, which included osmotic pressure (5% NaCl), varying pH levels (4.5, 7.0, and 9.5), and different temperatures (25 °C, 30 °C, and 37 °C). The cells were cultured with an initial optical density (OD_600_) of 0.1 and incubated for a further 24 h. 

### 2.2. Quantification of 2-PE

2-PE produced in bacteria cultured under different conditions was extracted using n-heptane at a ratio of 1:1. The conditions for the quantification of 2-PE using gas chromatography (GC) are as follows: a gas phase instrument equipped with an HP-Innowax capillary column (30 m × 0.25 mm × 0.25 μm) was used. The temperature procedure consisted of a 1 min hold at 50 °C, followed by a ramp from 50 °C to 120 °C at a rate of 15 °C/min, and finally a ramp from 120 °C to 240 °C at a rate of 20 °C/min for 10 min. Nitrogen (N_2_) was used as the carrier gas, and a flame ionization detector (FID) was employed. The gasification chamber was maintained at a temperature of 250 °C and the test chamber at 280 °C [2,8].

### 2.3. Quantification of IAA

The production of IAA of *Enterobacter* sp. CGMCC 5087 was quantitatively measured using Salkowski’s method. The specific method is as follows: A total 150 µL of bacterial solution was centrifuged at 13,000 rpm for 2 min. Then, 100 µL of the supernatant was transferred to a new 1.5 mL EP tube. The supernatant was mixed with 200 µL of Salkowski’s reagent (1 mL of 0.5 µM ferric chloride solution was added to 50 mL of 35% perchloric acid and thoroughly mixed). The mixture was allowed to react in the dark at room temperature for 20–25 min. After the reaction, the absorbance was measured at 530 nm using a microplate reader. The IAA concentrations of the samples were determined using an IAA standard curve for calibration [8,29].

### 2.4. RNA Isolation and Quantitative Real-Time RT-PCR

The primers used in this study are listed in Table S1. The previously described method was used with minor modification [8]. Briefly, the overnight culture of *Enterobacter* sp. CGMCC 5087 was diluted to an OD_600_ of 0.1 in different medium cultured in the aforementioned stress conditions. The culture was then incubated at 37 °C and 180 rpm for 12 h. RNA extraction was performed using the Flying Shark^®^ Plus Bacteria RNA Kit (gDNA-Filter) (Nobele, Beijing, China; RNE22) following the manufacturer’s instructions. cDNA was synthesized by HiScript^®^ III RT SuperMix for qPCR (+gDNA wiper) (Vazyme, Nanjing, China; R323-01) using total RNA as template. Gene expression was quantified through quantification using RT-qPCR with ChamQ Universal SYBR qPCR Master Mix (Vazyme, Nanjing, China; Q711) using a LightCycler^®^R480 Sequence Detection System (Roche, Bedford, MA, USA). The expression level of *rpoD* was detected and used as an internal reference [8].

### 2.5. Homology Modeling and Structure Optimization

Protein sequence for protein KDC4427 originated from *Enterobacter* sp. CGMCC 5087 was retrieved from the NCBI database with accession number WP_109845400 [30]. No 3D crystal structure was determined, and homology modeling for IPDC was conducted by Modeller software 10.5 [31]. As shown in Figure S1A, the crystal structure (PDB ID: 1OVM) [30] of IPDC from *Enterobacter cloacae* with sequence identity 94.32% was selected as modeling template. One > 10 ns position-constrained MD simulation was performed to eliminate the steric conflicts to achieve the optimized protein structure.

### 2.6. Molecular Docking of Protein KDC4427 to PPA/IPyA

Molecular docking process was conducted by software Autodock 4.2 [32] to predict the binding modes of KDC4427 to PPA or IPyA. The ligands PPA and IPyA were firstly sketched using ChemDraw 12.0 and then imported into the software MOE version 2019 [33] for 3D protonation and energy minimization operation. Then, the chemical structure of PPA or IPyA was saved as the Mol2 format. The binding pocket was resigned for rigid-body docking. Pose clustering was employed and 100 conformations were obtained for PPA or IPyA. Finally, the docking pose with largest pose clustering and the lowest binding energy was selected for further analysis.

### 2.7. Molecular Dynamics Simulation

In order to check the structural and thermodynamic and details of tertiary changes, one > 10 ns MD simulation for KDC4427/PPA or KDC4427/IPyA complex under each stress condition was performed using the software Amber 20 [34].

The KDC4427/PPA or KDC4427/IPyA complex was solvated within the TIP3P water model cubic box allowing a minimum of 10 Å marginal distance from each heavy atom of protein. The ligand and protein were parameterized with the force field parameters GAFF and AMBER99SB, respectively. The protein KDC4427 residues were assigned standard ionization states, considering physiological conditions at pH 7.0. Subsequently, the entire complex was neutralized by adding an appropriate number of Na^+^ ions (13) using the Monte-Carlo ion-placing method. A force constant of −1000 kJ/mol was applied to restrain all heavy atoms on KDC4427, and the MD simulation of the head pyrophosphate group was performed in three stages. The PPA or IPyA hydrophobic groups were freely optimized with no force restrained within MD simulations. The initial optimization of each system’s geometry began with 5000 iterations (5 ps) using the steepest descent algorithm. This was followed by a two-staged equilibration simulation, where the system was conditioned for 100,000 iterations (100 ps) at each stage. In the first equilibration stage, the system was maintained under a constant Number of particles, Volume, and Temperature (NVT) ensemble. The temperature was regulated within the 3D box using the Berendsen temperature coupling method. Subsequently, the second equilibration stage was conducted under a constant Number of particles, Pressure, and Temperature (NPT) ensemble, at 1 atm and 303.15 K, using the Parrinello–Rahman barostat. The Root Mean Square Deviation (RMSD) values for KDC4427/PPA or KDC4427/IPyA complex under each condition was checked until the MD simulation reached equilibrium state. Binding mode and binding free energy between KDC4427 and PPA or IPyA were extracted for detailed analysis.

## 3. Results and Discussion

### 3.1. Effect of Osmotic Stress on the Growth and the Products Synthesis

**Figure 1:**
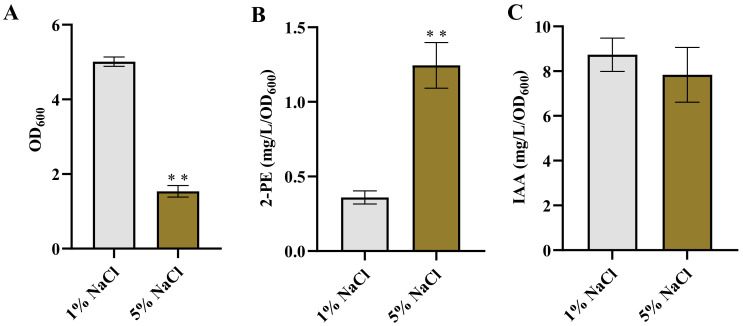
The growth and production of 2-PE and IAA in *Enterobacter* sp. CGMCC 5087 under osmotic stress. (**A**) The growth of *Enterobacter* sp. CGMCC 5087 under 1% or 5% NaCl stress conditions at 24 h. (**B**) The effect of 5% NaCl on production of 2-PE. The concentration of 2-PE was measured under normal (1%) or 5% NaCl stress at 24 h. (**C**) The effect of 5% NaCl on production of IAA. The concentration of IAA was measured under normal or 5% NaCl stress at 24 h. The data shown are means ± SD (*n* = 3). Significant differences were analyzed by two-sample *t*-test, and *p* < 0.05 was considered statistically significant. ** *p* < 0.01.

To investigate the effect of osmotic stress on the production of 2-PE and IAA in *Enterobacter* sp. CGMCC 5087, the bacteria was cultured in LB supplemented with 5% NaCl. Under the presence of 5% NaCl stress, bacterial growth was significantly inhibited, whereas its ability to synthesize 2-PE was significantly enhanced, and the ability to synthesize IAA was slightly reduced with no significant difference compared to the control (Figure 1). The growth inhibition caused by 5% NaCl may be due to the disruption of osmotic balance resulting from the high salt environment [35,36,37]. Penetration pressure has a certain influence on the ion homeostasis of bacteria, and bacteria have developed complex mechanisms for ion homeostasis, mainly involving Na^+^. Na^+^ can passively flow into cells in response to external high concentrations of NaCl or be actively taken up through cotransport with certain types of compatible solutes to accumulate Na^+^. Cells mainly actively maintain low cytoplasmic concentrations of toxic Na^+^ ions by the activity of Na^+^ antiporters. When Na^+^ accumulates excessively without proper regulation, it causes damage to the growth and metabolism of bacteria [36,38]. In addition, although the high concentration of salt environment inhibited the growth of *Enterobacter* sp. CGMCC 5087, it did not completely deactivate it. This may be related to the accumulation of compatible solutes (such as proline, trehalose, etc.). Compatible solutes can counteract some of the harmful effects of osmotic pressure dehydration. While the accumulation of compatible solutes consumes energy, it provides a flexible means for bacterial cells to adapt to a wide range of environmental salinity and osmotic pressure [36,39,40]. However, despite the growth inhibition, the bacteria cells exhibited a comparable or even enhanced ability to synthesize these metabolic products compared to the control. These results indicate that *Enterobacter* sp. CGMCC 5087 could be adapted to changes in high osmotic environments.

It was reported that the production of 3-methylthiopropanol and 2-PE were increased in *Zygosaccharomyces rouxii* and Z. *rouxii* 3-2, while the synthesis of isoamyl acetate was inhibited under high salt conditions [41]. *Burkholderia* sp. MTCC 12259 produces IAA and extracellular polymeric substances (EPS), which are important factors triggering plant growth under salt stress. Interestingly, both IAA and EPS production are maximized in the absence of salt stress and gradually decrease as salt concentrations increase. However, the production of proline, functioning as an osmoprotectant, is enhanced with increasing salt concentration [42]. Meanwhile, bacteria also respond to adverse environments by adjusting their energy metabolism. In a high osmotic pressure environment with 5% NaCl, the accumulation of trehalose in *E. coli* BW25113 was promoted, and the accumulation of trehalose played a crucial role in this high osmotic stress response [43]. Therefore, the enhancement or maintenance of bacterial synthesis capability under salt stress can be attributed to the activation of adaptive metabolic pathways or enzyme activities in response to the high salt environment during the stress adaptation of the bacterial cells [44,45,46,47]. However, further research is required to elucidate the specific response mechanisms and molecular basis underlying the enhanced synthesis ability.

### 3.2. Effect of pH on Growth and Products Synthesis

**Figure 2:**
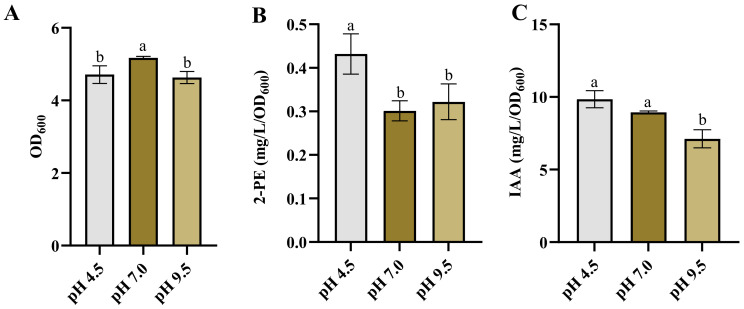
The growth and production of 2-PE and IAA in *Enterobacter* sp. CGMCC 5087 under different pH. (**A**) The growth of *Enterobacter* sp. CGMCC 5087 under pH 4.5, pH 7.0, and pH 9.5 stress conditions at 24 h. (**B**) The effect of pH 4.5, pH 7.0, and pH 9.5 on production of 2-PE. The concentration of 2-PE was measured under pH 4.5, pH 7.0, or pH 9.5 at 24 h. (**C**) The effect of pH 4.5, pH 7.0, and pH 9.5 on production of IAA. The concentration of IAA was measured under pH 4.5, pH 7.0, or pH 9.5 at 24 h. The data shown are means ± SD (*n* = 3). Different letters a and b indicate a statistically significant difference (Duncan’s multiple range test, *p* < 0.05).

The influence of pH on the production of various end-products has been well-documented [6,48,49,50]. In this study, we examined the effect of pH on 2-PE and IAA production. The growth of *Enterobacter* sp. CGMCC 5087 was inhibited in environments with pH values of 4.5 and 9.5 compared to those with pH 7.0 (Figure 2A). However, the acidic culture environment at pH 4.5 significantly enhanced the synthesis of 2-PE, while there was no significant difference in the synthesis capability of IAA compared with that of the neutral condition (Figure 2B,C). On the other hand, under alkaline stress conditions at pH 9.5, there was no significant difference in the synthesis capability of 2-PE compared to the neutral condition, but the synthesis capability of IAA was reduced (Figure 2B,C). This highlights that although acidic conditions inhibit the growth of *Enterobacter* sp. CGMCC 5087, they can promote the synthesis of metabolic products. This also implies that utilizing this characteristic during the fermentation process of 2-PE can enhance the yield. 

The synthesis of 2-PE and IAA is dependent on α-ketoacid decarboxylase KDC4427, and the alteration in synthesis capability is inseparable from the influence of KDC4427 activity. The research indicated that the activity of KDC4427 is highest at pH 6 to 6.5 [7]. However, our results showed that the production of 2-PE was highest at pH 4.5. The inconsistency in this performance may be related to the homeostasis within the bacteria. The maintenance of pH homeostasis is the result of the interaction of multiple transport systems. Microorganisms have evolved different strategies to resist acid stress to maintain pH homeostasis. Some yeasts and bacteria keep a relatively stable and neutral intracellular pH in the presence of constantly changing extracellular pH [51]. Under acidic conditions at pH 4.2, *E. coli* upregulated the key genes of the two-component system CpxRA, leading to an increase in the production of unsaturated fatty acids. This further reduced membrane fluidity and F_0_F_1_-ATPase activity, helping the cell to approach a neutral intracellular steady state [52]. Under acidic or alkaline conditions, bacteria face differences in pH values inside and outside the cell, which may lead to changes in protein structure and, therefore, affect enzyme activity [53]. Similarly to methanogens, extreme pH stress significantly inhibited metabolic pathways and cellular regulation. pH stress reduced the activities of most enzymes associated with methanogens, including acetate kinase, formylmethanofuran dehydrogenase, and so on. It also hindered the transfer of electron carriers and inhibited ATP synthesis to regulate energy metabolism. However, under alkaline conditions, the levels of EPS and total EPS proteins were generally increased [54]. Therefore, this phenomenon also might arise as a result of a combination of metabolic alterations in the bacteria cell and variations in gene expression. Studies have shown that bacteria are capable of enhancing the utilization rate of non-preferred substrates and adapting to environmental stress, despite a decrease in enzyme activity [55]. This adaptability can lead to modifications in gene expression, enzyme activity, metabolic pathways, and substrate utilization efficiency, improving synthesis ability in stressful environments [56,57,58,59,60]. 

### 3.3. Effect of the Temperature on the Growth and the Products Synthesis

**Figure 3:**
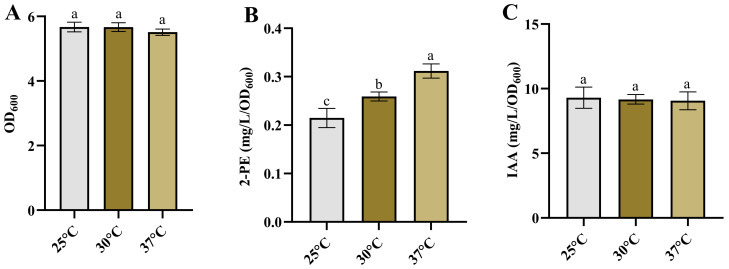
The growth and production of 2-PE and IAA in *Enterobacter* sp. CGMCC 5087 under different temperature. (**A**) The growth of *Enterobacter* sp. CGMCC 5087 under 25 °C, 30 °C, and 37 °C conditions at 24 h. (**B**) The effect of 25 °C, 30 °C, and 37 °C on production of 2-PE. The concentration of 2-PE was measured under 25 °C, 30 °C, and 37 °C conditions at 24 h. (**C**) The effect of 25 °C, 30 °C, and 37 °C on production of IAA. The concentration of IAA was measured under 25 °C, 30 °C, and 37 °C conditions at 24 h. The data shown are means ± SD (*n* = 3). Different letters a to c indicate statistically significant difference. Same letters indicate statistically insignificant difference (Duncan’s multiple range test, *p* < 0.05).

Temperature plays a crucial role in the growth, metabolism, and subsequent product synthesis of bacteria [61,62,63,64]. Some bacteria are sensitive to temperature changes, with alterations in temperature having a significant impact on their product synthesis [6,63,65]. Therefore, we investigated the growth and synthesis of 2-PE and IAA in *Enterobacter* sp. CGMCC 5087 under different temperature conditions. The results indicated that altering the temperature between 25 °C and 37 °C did not influence the growth and synthesis capacity of IAA (Figure 3A,C). However, within this temperature range, the synthesis ability of 2-PE gradually increased as the temperature rose (Figure 3B). These results suggest that *Enterobacter* sp. CGMCC 5087 possesses a wide temperature range adaptability in growth and IAA synthesis. Therefore, temperature is not a critical factor in the fermentation production of IAA, and can be fermented within the temperature range of 25 °C to 37 °C. However, changes in temperature are closely linked to the synthesis of 2-PE. The PPDC activity of KDC4427 at 25 °C is lower compared with that at temperatures of 30 °C and 37 °C; this is consistent with our experimental results [7]. Nevertheless, the synthetic capacity of IAA did not significantly change with temperature variation. This may be due to the variation in expression and IPDC activity of KDC4427. In order to explore the mechanism underlying this phenomenon, gene expression and enzyme activity analysis were performed on KDC4427 under the aforementioned stress conditions.

### 3.4. The Analysis of KDC4427 Gene Expression in Different Conditions

**Figure 4:**
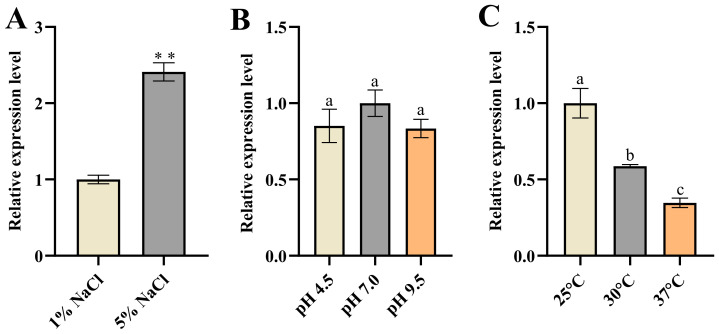
The expression of *KDC4427* under different stress conditions. (**A**) The expression of *KDC4427* under 1% NaCl and 5% NaCl stress conditions. Significant differences were analyzed by two-sample *t*-test, and *p* < 0.05 was considered statistically significant; ** *p* < 0.01. (**B**) The expression of *KDC4427* under pH 4.5, pH 7.0, and pH 9.5 stress conditions. (**C**) The expression of *KDC4427* under 25 °C, 30 °C, and 37 °C conditions. The data shown are means ± SD (*n* = 3). Different letters a to c indicate statistically significant difference. Same letters indicate statistically insignificant difference (Duncan’s multiple range test, *p* < 0.05).

We investigated the expression of *KDC4427* under these stress conditions. It was found that the gene expression under 5% NaCl was enhanced compared with that under 1% NaCl (Figure 4A). The result implies that bacteria enhance their synthesis capacity of 2-PE under osmotic pressure stress by upregulating *KDC4427* expression. However, increasing the expression of *KDC4427* did not affect the production of IAA (Figure 1C and Figure 4A). Analysis of *KDC4427* expression under pH stress conditions revealed no significant changes in mRNA abundance for the three pH levels mentioned above (Figure 4B). However, KDC4427 exhibits optimal enzyme activity at pH 6 to 6.5, and enzyme activity is significantly reduced at pH levels below 5 or above 8 [7]. These data suggest that pH stress did not affect the expression of the *KDC4427* but regulated the activity of the KDC4427 through other pathways, thereby influencing the synthesis of 2-PE and IAA.

Finally, the results showed that the expression of *KDC4427* decreased as the temperature increased (Figure 4C). Previous studies have shown that the PPDC activity of KDC4427 increased within the range of 25 °C to 37 °C [7]. Our results showed that the synthesis of 2-PE was enhanced as temperature rose (Figure 3B). These data suggested that the catalytic activity of KDC4427 played a more important role in the synthesis of 2-PE. The elevated expression of *KDC4427* under low-temperature conditions did not affect the synthesis ability of IAA (Figure 3C and Figure 4C). This also implied that the variations in KDC4427 enzyme activity have a crucial role in the synthesis of IAA.

### 3.5. Effect of Changing Conditions on Enzyme Catalysis

In vitro molecular simulation experiments provided a reduced-scale observation and data to help understand the reactions of bacterial cells under different conditions, inferring their potential physiological and biochemical responses in specific environments. They could not directly indicate the in vivo conditions, but the simulated results from in vitro experiments still offered some useful clues and information from another perspective.

**Figure 5:**
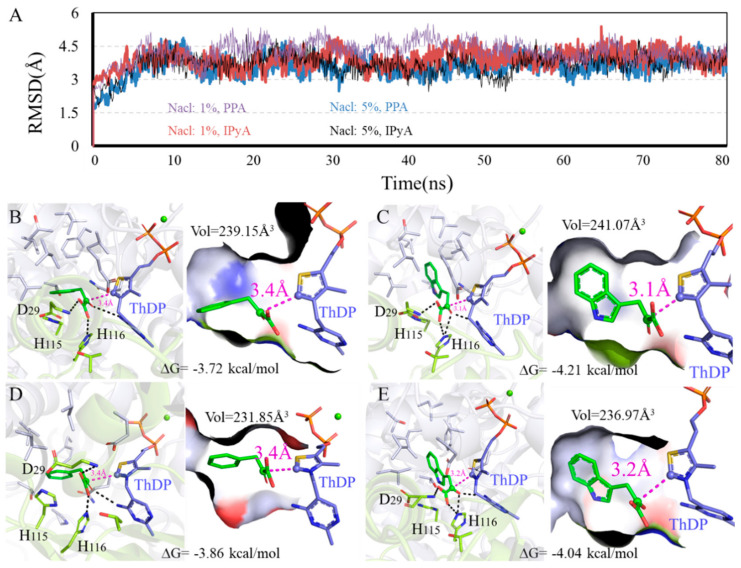
Binding modes difference of KDC4427 with the substrates PPA or IPyA at different NaCl content (1% and 5%). (**A**) Average structure of KDC4427/PPA or KDC4427/IPyA at different NaCl content was extracted from 80 ns MD simulations. (**B**) Interaction of KDC4427/PPA at 5% NaCl. (**C**) Interaction of KDC4427/IPyA at 5% NaCl. (**D**) Interaction of KDC4427/PPA at 1% NaCl. (**E**) Interaction of KDC4427/IPyA at 1% NaCl. The distance *d_αC-C-_* between αC atom (PPA and IPyA) and C^-^ atom (ThDP) was measured and colored by magenta dashed line. Hydrogen bond was denoted by black dashed line.

**Figure 6:**
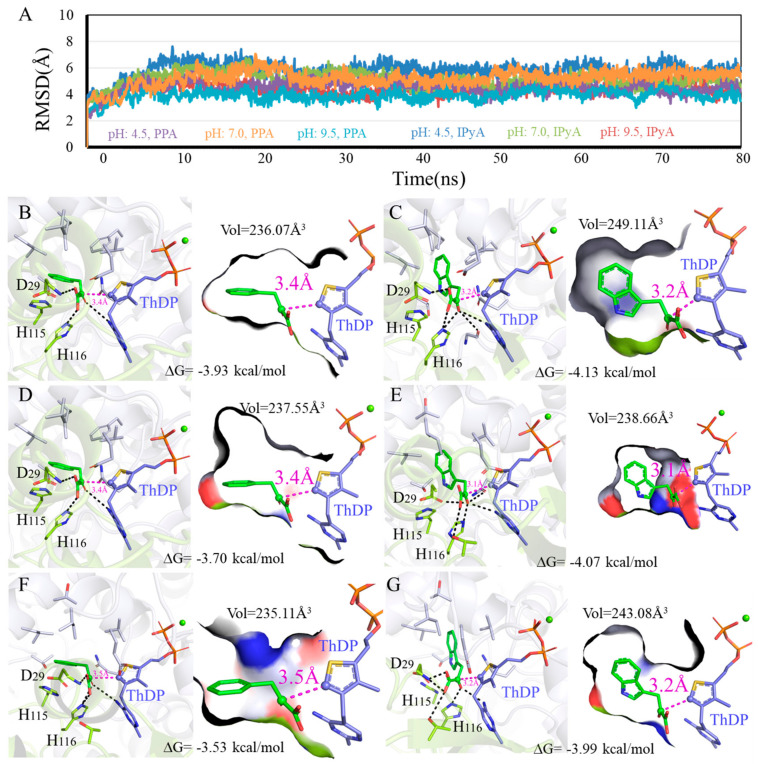
Binding modes difference of KDC4427 with the substrates PPA or IPyA at different pH values (4.5, 7.0, and 9.5). (**A**) Average structure of KDC4427/PPA or KDC4427/IPyA at different pH content was extracted from 80 ns MD simulations. (**B**) Interaction of KDC4427/PPA at pH 4.5. (**C**) Interaction of KDC4427/IPyA at pH 4.5. (**D**) Interaction of KDC4427/PPA at pH 7.0. (**E**) Interaction of KDC4427/IPyA at pH 7.0. (**F**) Interaction of KDC4427/PPA at pH 9.5. (**G**) Interaction of KDC4427/IPyA at pH 9.5. The distance *d_αC-C-_* between αC atom (PPA and IPyA) and C^-^ atom (ThDP) was measured and colored by magenta dashed line. Hydrogen bond was denoted by black dashed line.

**Figure 7:**
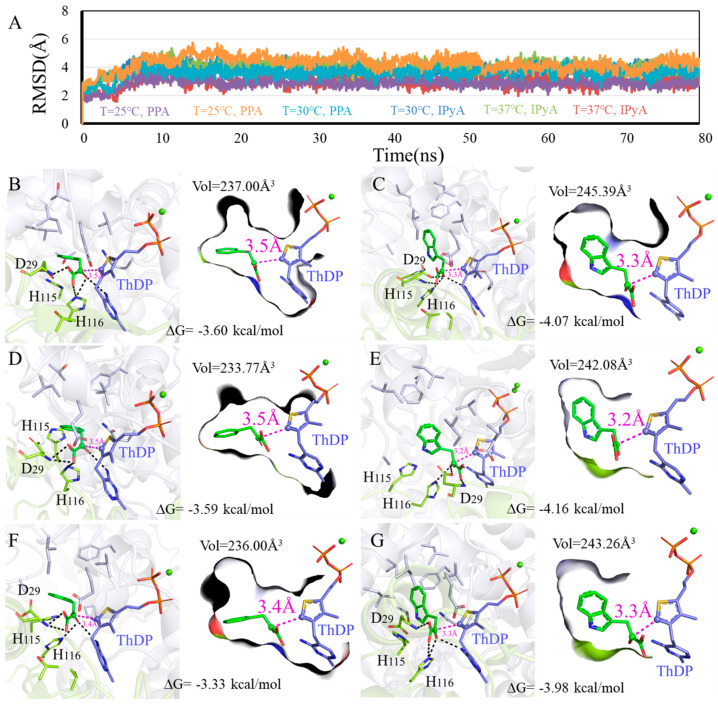
Binding modes difference of KDC4427 with the substrates PPA or IPyA at different temperature (25 °C, 30 °C, and 37 °C). (**A**) Average structure of KDC4427/PPA or KDC4427/IPyA at different temperature was extracted from 80 ns MD simulations. (**B**) Interaction of KDC4427/PPA at 25 °C. (**C**) Interaction of KDC4427/IPyA at 25 °C. (**D**) Interaction of KDC4427/PPA at 30 °C. (**E**) Interaction of KDC4427/IPyA at 30 °C. (**F**) Interaction of KDC4427/PPA at 37 °C. (**G**) Interaction of KDC4427/IPyA at 37 °C. The distance *d_αC-C-_* between αC atom (PPA and IPyA) and C^-^ atom (ThDP) was measured and colored by magenta dashed line. Hydrogen bond was denoted by black dashed line.

To explore how the different conditions affect the enzyme activity of KDC4427, the detailed interactions between KDC4427 and PPA or IPyA, molecular modeling, and molecular docking analysis were conducted and systematically analyzed. The modeling structure and binding modes of KDC4427/PPA or KDC4427/IPyA are shown in Figures S1 and S2. > 90 ns MD simulations under different NaCl, pH, and temperature conditions were performed to investigate the structure differences of KDC4427-PPA and KDC4427-IPyA complexes in the physicochemical environment. Given the geometry of catalytic center, binding free energies, binding cavity volume, and the distance *d_αC-C-_* between αC atom (PPA and IPyA) and C-atom (ThDP) were monitored to quantify the subtle variations in catalytic activities. The in silico predictions and binding modes of influence under these different conditions and corresponding catalytic activities are collected in Figure 5, Figure 6 and Figure 7.

#### 3.5.1. Influence of Different NaCl Content on Enzyme Catalysis

RMSD values and binding modes of representative structures from the balanced simulations at different NaCl content are shown in Figure 5. Figure 5B,D and Figure 5C,E show the calculated binding modes difference of PPA and IPyA to KDC4427 at 1% NaCl and 5% NaCl content environment. As shown in Figure 5B,D, the PPA both showed a relatively spacious appearance with the binding cavity, as crowded spaces at 239.15 Å^3^ and 231.85 Å^3^ were observed in a 1% and 5% NaCl content environment. Despite the same *d_αC-C-_* distance values at 3.4 Å, PPA possesses a lower binding energy −3.86 kcal/mol under 5% NaCl than that of -3.72 kcal/mol under 1% NaCl content. As shown in Figure 5C,E, the molecule IPyA could occupy almost the same binding cavity as the volume data were filled and amplified to 241.07 Å^3^ and 236.97 Å^3^, respectively. However, IPyA owns a shorter *d_αC-C-_* distance with protein under 1% (3.1 Å) than that under 5% NaCl (3.2 Å) content. In addition, little differences in activation free energy were also observed at 1% NaCl (−4.21 kcal/mol), with at most 0.17 kcal/mol improvement, compared with that at 5% NaCl content (−4.04 kcal/mol). Comprehensive comparing analysis of calculation results showed that KDC4427 had a better catalytic capacity for PPA at 5% than 1% NaCl content, which was consistent with the experimental results and for IPyA at 1% than 5% NaCl content. However, our experimental results showed that the ability to synthesize IAA under 1% and 5% NaCl conditions was the same. This indicates that under 5% NaCl stress, besides KDC4427 participating in the synthesis of IAA, other metabolic pathways were involved in maintaining the stability of IAA synthesis. It was speculated that intracellular compatible solutes may have played a certain role in maintaining and regulating IAA synthesis.

#### 3.5.2. Influence of Different pH on Enzyme Catalysis

To investigate the effects of different pH on enzyme catalysis, we performed 3D variability analysis of the binding modes data (Figure 6). As shown in Figure 6B,D,F, contacting compactness under pH 4.5, pH 7.0, and pH 9.5 environments was almost same at 236.07 Å^3^, 237.55 Å^3^, and 235.11 Å^3^, respectively. However, the molecule PPA exhibited an obvious higher binding affinity under pH 4.5 (−3.93 kcal/mol) and pH 7.0 (−3.70 kcal/mol) than pH 9.5 (−3.53 kcal/mol). In addition, the distance *d_αC-C_*_-_ values for PPA were both 3.4 Å at pH 4.5 and pH 7.0, which was smaller compared with that under a pH 9.5 environment. As shown in Figure 6C,E,G, within the effective binding poses, the αC atom on IPyA molecule was at 3.1 Å under the pH 7.0 condition and closer than 3.2 Å under both pH 4.5 and pH 9.5. Binding affinities for KDC4427/IPyA system reached −4.13 kcal/mol at pH 4.5, −4.07 kcal/mol at pH 7.0, and −3.99 kcal/mol at pH 9.5, presenting the opposite trend with the volume values. The above calculation results showed that the catalytic capabilities for KDC4427 to PPA and IPyA were pH 7.0 > pH 4.5 ≈ pH 9.5 and pH 4.5 ≈ pH 7.0 > pH 9.5, respectively. The results showed that the IPDC activity of KDC4427 was consistent with the results of IAA synthesis under different pH conditions. However, the synthesis ability of 2-PE at pH 4.5 is stronger than that at pH 7.0 and pH 9.5, and there was no significant difference in *KDC4427* expression, indicating that the synthesis of 2-PE was still influenced by other factors. In addition, the in vitro simulation results also indicated that *Enterobacter* sp. CGMCC 5087 prefers a neutral environment (pH 7.0) for product synthesis. The ability to synthesize 2-PE was stronger at pH 4.5, and the IAA synthesis capability was the same as at pH 7.0. It was speculated that under acidic stress, the bacteria might maintain their own growth and enhance product synthesis abilities by balancing intracellular pH and regulating their own homeostasis.

#### 3.5.3. Influence of Different Temperature on Enzyme Catalysis

As shown in Figure 7A, the systems all reached equilibrium after 15 ns, and binding modes analysis revealed that PPA and IPyA can be buried into the binding cavity in geometric configurations with −4.16~−3.33 kcal/mol binding free energy. The smaller size for PPA hastened flexible conformations to the binding cavity, resulted a decrease in binding affinities (25 °C: −3.60 kcal/mol, 30 °C: −3.59 kcal/mol, and 37 °C: −3.33 kcal/mol), and collapsed the binding cavity (25 °C: 237.00Å^3^, 30 °C: 233.77Å^3^, and 37 °C: 236.00Å^3^) (Figure 7B,D,F). Although the PPA at 37 °C owns the lowest binding affinity -3.33 kcal/mol, it possesses the shortest *d_αC-C_*_-_ distance value of 3.4 Å. Thus, KDC4427 presents better catalytic efficiency to PPA at 37 °C, which is consistent with the experimental data. Under temperatures of 25 °C and 30 °C, the hydrogen bonds network with residues H116 and D29 was observed in the binding pocket and relatively intense binding patterns (−4.07 kcal/mol and −4.16 kcal/mol) were observed for IPyA (Figure 7C,E). The loose interaction occurred at temperature 37 °C as the binding affinity, cavity volume, and distance *d_αC-C-_* for IPyA reached −3.98 kcal/mol, 243.26 Å^3^, and 3.3 Å, respectively (Figure 7G). Despite different binding affinity and geometric characteristics, the enzyme also exhibited better catalytic capability to IPyA under a 30 °C environment. However, our results indicated that temperatures ranging from 25 to 37 °C had no impact on the synthesis ability of IAA. This suggested that, in addition to the involvement of KDC4427 in the synthesis, other regulatory mechanisms also played a collective role in influencing the synthesis of IAA.

## 4. Conclusions

The results of this study demonstrate that *Enterobacter* sp. CGMCC 5087 exhibits good adaptability to osmotic pressure, pH stress, and different temperature growth environments. The synthesis ability of 2-PE was enhanced under stress conditions of 5% NaCl and pH 4.5. However, there was no significant alteration in the synthesis ability of IAA. In an alkaline environment with a pH of 9.5, there was no significant alteration in the synthesis capability of 2-PE but a reduction in the synthesis capability of IAA was observed. In addition, varying the temperature within an appropriate range did not significantly affect the synthesis ability of IAA but did have a significant impact on the synthesis of 2-PE. As the temperature increased, the synthesis ability of 2-PE was enhanced. Additionally, we conducted expression analysis of the key gene *KDC4427* both in the production of 2-PE and IAA. Under 5% NaCl stress, the expression of *KDC4427* was significantly increased while there were no differences under pH conditions. However, as the temperature increased, the expression level of *KDC4427* gradually decreased. Finally, in silico methods combining molecular docking and MD simulations predicted the catalytic mechanism difference under these conditions. The results indicated that the catalytic efficiency can be characterized by the binding cavity volume, binding free energy, and distance *d_αC-C-_* parameters. Therefore, in light of above results, optimal conditions can be adjusted during the fermentation process of 2-PE and IAA. For example, during the fermentation of 2-PE, the culture temperature was appropriately increased, creating a slightly acidic fermentation environment or increasing osmotic pressure to enhance the yield of the target product. Moreover, the balance between bacteria growth and production should be carefully considered in the different conditions to achieve the optimal yield of target product.

In summary, these results reveal that *Enterobacter* sp. CGMCC 5087 can regulate the synthesis of 2-PE and IAA by regulating the expression of the key gene *KDC4427*, as well as its enzyme activity depending on environment conditions. Furthermore, the exploration of 2-PE and IAA synthesis under stress conditions in *Enterobacter* sp. CGMCC 5087 provides a theoretical basis and strategies for future fermentation production.

## Data Availability

Data are contained within the article.

## Supplementary Materials

The following supporting information can be downloaded at: https://www.mdpi.com/article/10.3390/microorganisms12040663/s1, Table S1: Primers used in this study. Figure S1: Molecular basis of KDC4427 for catalytic function. Figure S2: The best molecular docking poses of KDC4427 with the substrates PPA or IPyA at catalytic center.
