# Supplementary material for: Effects of Environmental Stresses on Synthesis of 2-Phenylethanol and IAA by Enterobacter sp. CGMCC 5087
Source: Microorganisms. 2024 Mar 26;12(4):663. doi: 10.3390/microorganisms12040663 (PMC11052032; doi:10.3390/microorganisms12040663)
Supplement: Supplementary file 1 [file microorganisms-12-00663-s001.zip › supplementary-microorganisms-2893618.pdf]

Supplementary Materials

Effects of environmental stresses on synthesis of 2-phenylethanol and IAA by *Enterobacter* sp. CGMCC 5087

Ke Li [1,2,3,4,†], Senbiao Fang [2,3,4,†], Xiao Zhang [2,3,4], Xiaodi Wei [1,2,3,4], Pingle Wu [1,2,3,4], Rong Zheng[1,*], Lijuan Liu [2,3,4,*] and Haibo Zhang[2,3,4,*].

[1] College of Life Science and Technology, Inner Mongolia Normal University, Hohhot 010022, China

[2] Qingdao Institute of Bioenergy and Bioprocess Technology, Chinese Academy of Sciences, Qingdao 266101, China

[3] Shandong Energy Institute, Qingdao 266101, China

[4] Qingdao New Energy Shandong Laboratory, Qingdao 266101, China

[*] Corresponding authors

[†] These authors contributed equally

Table S1. Primers used in this study

Primer name	DNA sequence	source
q*rpoD*-F	CCCGTGAAGCGAAAGTCCT	[1]
q*rpoD*-R	CTTCAGAGCGGCTTGGATGA	
q*KDC4427*-F	CGTTCCGGTGCTGCATATTG	
q*KDC4427*-R	CGTTCCGGTGCTGCATATTG	

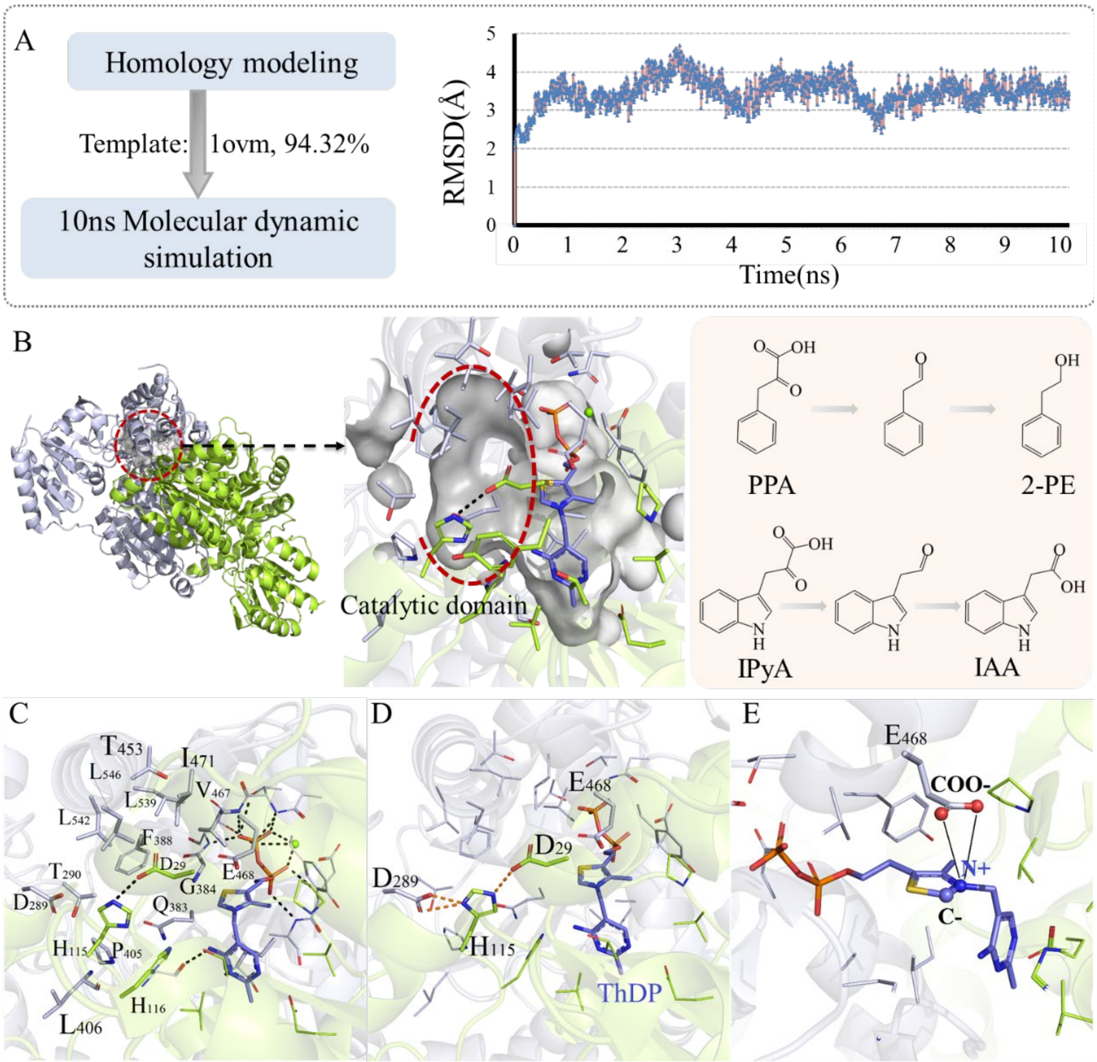

Figure S1. Molecular basis of KDC4427 for catalytic function. (A) 3D structure was obtained by homology modeling (template: IPDC, sequence identity 94.32%, PDB ID: 1OVM) and optimized by 10ns molecular dynamics simulations. (B) In the dimer structure, two chains composed the catalytic center at the binding interface. (C-D) The catalytic center was formed by 6 hydrophilic and 14 hydrophobic residues (C) with highlighted triad members D29-H115-E468 (D). (E) The amino acid E468 plays a decisive role in maintaining the negative state of C^- atom on ThDP.

In this project, the crystal structure of indole-3-pyruvate decarboxylase (IPDC) with sequence identity 94.32% (PDB ID: 1OVM) was selected for homology modeling and > 10ns MD simulations (Figure S1A). Two identical protein chains form the catalytic pocket that can catalyze the decarboxylation of PPA and IPyA to phenylacetaldehyde and indoleacetaldehyde, respectively (Figure S1B). One total number of 20 residues forming the catalytic region play crucial roles in the catalytic mechanism. 14 hydrophobic residues including F388, P405, L406, I471, L539, L542 and etc., formed one small hydrophobic binding cavity, which plays a role in

accommodating substrate conformation and specific catalysis. In addition, 6 hydrophilic residues H115, H116, D29, D289, T290 and Q383 (Figure S1C) were observed in the binding pocket, where 3 residues form the catalytic triad D29-H115-E468 (Figure S1D). It is worth noting that the residue E468, above the thiazole ring of ThDP on coenzyme thiamine pyrophosphate (ThDP) domain, plays important role in maintaining the positive N^+ and negative C^- states (Figure S1E). It is speculated that any structural interference at this amino acid site E468 or interaction mode between ThDP and the substrate will cause a decrease or even loss of catalytic activity.

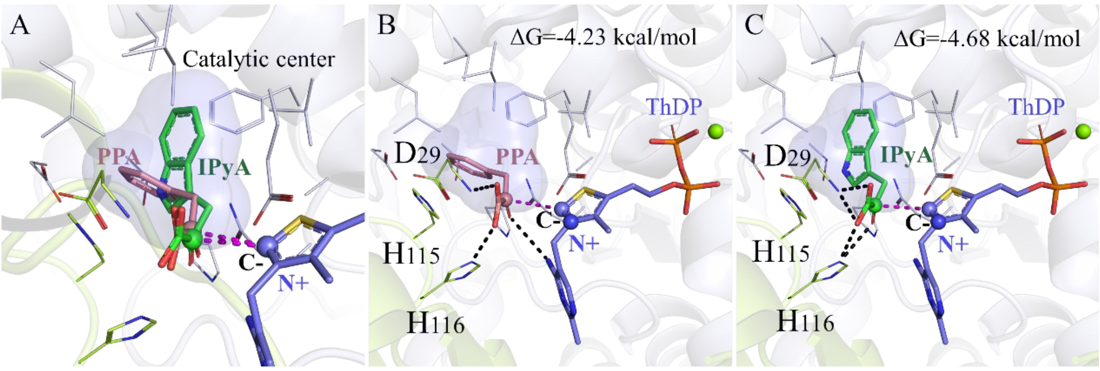

Figure S2. The best molecular docking poses of KDC4427 with the substrates PPA or IPyA at catalytic center. (A) Binding poses superimposition of PPA and IPyA with enzyme. (B) Interaction modes of KDC4427/PPA complex with binding energy at -4.23 kcal/mol. (C) Interaction modes of KDC4427/IPyA complex with binding energy at -4.68 kcal/mol.

In order to explain detailed interactions between KDC4427 and PPA or IPyA, molecular docking analysis were conducted and systematically analyzed. The best docking poses of PPA and IPyA indicated that the catalytic center contributed a small geometric pocket which can accommodate about two benzene rings (Figure S2A). It should be noted that the ligand PPA has a smaller benzene ring and thus can't occupy the entire space of catalytic center, resulting in weaker binding strength at -4.23 kcal/mol. The ligand PPA was embedded in hydrophobic interaction with surrounding nonpolar residues and at last stabilized by hydrogen bonding with residues D29, H116 and ThDP (Figure S2B). On the contrary, the molecule IPyA possesses a bigger benzpyrole, allowing it to be more completely packaged by hydrophobic residues, and also can keep more intensive hydrogen bonds with polar residues D29 and H116 (Figure

S2C). Furthermore, the carboxylate chemical functional groups of PPA or IPyA need to extend towards the E468 side chain in order to be catalyzed, and the more appropriate the orientation, the higher the catalytic efficiency. The specific binding of αC atom (PPA and IPyA) to C^- atom (coenzyme factor ThDP) should be required to maintain the activated catalytic modes of KDC4427 to substrates.

References

1. Liu, L.; Chen, G.; Liu, J.; Bao, W.; Li, X.; Yang, K.; Shi, S.; Zhao, B.; Wang, Q.; Cao, X.; et al. Sequential production of secondary metabolites by one operon affects interspecies interactions in *Enterobacter* sp. CGMCC 5087. *The Innovation Life* **2023**, *1*, 100023.
